# Assessing the Effectiveness of the Aging Mastery Program

**DOI:** 10.3390/healthcare6020041

**Published:** 2018-05-07

**Authors:** Lisa Ferretti, Philip McCallion, Emily McDonald, Hayoung Kye, Angelica P. Herrera-Venson, James Firman

**Affiliations:** 1 School of Social Work, Temple University, Philadelphia, PA 19122, USA; lisa.ferretti@temple.edu; 2 National Council on Aging, Washington, DC 20009, USA; Emily.McDonald@ncoa.org (E.M.); Hayoung.kye@ncoa.org (H.K.); angelica.herrera-venson@ncoa.org (A.P.H.-V.); James.Firman@ncoa.org (J.F.)

**Keywords:** physical activity, advanced planning, aging, evidence-based

## Abstract

*Background*: Successful aging is best determined by active management and self-determination of one’s aging roadmap. Some individuals are ready to respond to these challenges, others may benefit from assistance that might be offered through an evidence-based intervention. The Aging Mastery Program^®^ (AMP) has been developed to meet these needs. *Method*: In a cross over design the intervention was tested in ten senior centers and aging network agencies looking at impacts upon general health and quality of life, patient activation, physical activity and advanced care planning. *Results*: There was a statistically significant (tested at a 0.05 level) level of improvement found in physical activity and advanced care planning. *Conclusion*: Findings support the program’s effectiveness and its value as an evidence-based intervention for older adult programming.

## 1. Introduction

In 2011 the oldest baby boomers started to turn 65, joining 40 million people in the United States aged 65 and older. There are projections this group will double to more than 89 million by 2050 with numbers aged 85 and older projected to increase more rapidly [1]. Changes in health and disability status, living arrangements, kinship networks and economic well-being are occurring. Over the past 30 years the health of older adults has improved with declines in rates of mortality and late onset disability and increases in improved ability to treat diseases and chronic illness, there is also increased prevalence of ongoing chronic conditions [1]. Half of all adults have one or more chronic conditions, one in four have two or more and rates are highest among those over 65 [2,3]. Older women, African-Americans and Latinos and those living in poverty are particularly at risk for added chronic conditions [4].

Quality of life is now an increasing consideration for older adults [5] in terms of (1) Material well-being—ability (often financial) to meet needs for basics such as food and shelter; (2) Physical well-being—the ability to perform basic activities of daily living and to live independently; (3) Social engagement—involvement with and support received from family, peers, community members and community organizations; and (4) Emotional well-being—mental and psychological wellness often tied to physical health and social support [5]. Each of these domains, therefore, is an area critical to experienced quality of life.

There is emerging evidence for the effectiveness of interventions targeting at least some of the outlined concerns. Examples of useful interventions are the Chronic Disease Self-Management Program supported by multiple randomized control trials completed with diverse populations [6], the Diabetes Prevention Program tested over extended periods of time [7] and interventions targeted at falls reduction, increased physical activity and management of specific chronic conditions supported by one time, randomized control and quasi-experimental studies. The more robust studies have helped establish the characteristics of successful programs: content related both to the purpose of the intervention and with stated underlying change process assumptions; manualized approaches; fidelity requirements; and standardized training [8]. However, the available interventions tend to target chronic conditions, address specific challenges such as falls; and offer skills in areas such as avoidance of financial abuse or building self-management readiness [9,10,11]. There is an absence of interventions targeting quality of life of older adults.

Another challenging issue is the extent to which the mechanics of the scale-up of interventions can be achieved so that the people most likely to benefit will be reached. Diffusion of innovation theory [12] suggests five characteristics of innovations that often determine the rate at which they are adopted: (1) relative advantage (both benefits and costs); (2) compatibility; (3) simplicity; (4) testability; and (5) observability. Many evidence-based community interventions were first designed for effectiveness and not for widespread implementation. There is a history of difficulty in bringing such interventions to scale [13]. A further complexity is that successful scaling of community-based health promotion programs often require adoption by at least three different types of stakeholders: consumer (participants), community or healthcare organizations (implementers) and government agencies or health systems/plans (payers). It is likely but largely untested, that interventions that speak directly to critical issues for older adults, particularly desires to age successfully, are most likely to be amenable to scalability.

Rowe and Kahn [14] described successful aging as having three critical components: absence or avoidance of disease and risk factors for disease, maintenance of physical and cognitive functioning and active engagement with life. Consideration of these issues begins from a belief that independence and self-determination are largely expressed and experienced in the middle adult and early aging years but because of changes in older years they come increasingly under threat. Continued independence and self-determination in older years reflects the person’s choices about where to live and in the management of major decisions about one’s life and care, when care is needed. One’s successful aging is best determined by individual actions [15]. Some individuals are ready to respond to these challenges, others may benefit from assistance that might be offered through an evidence-based intervention.

To date, there is no such intervention but preliminary work on the Aging Mastery Program^®^ (AMP) has supported such development and, consistent with recommended best practices [8], a manual and training and fidelity approaches have been developed; feasibility of delivery is established; and there is preliminary data on the program’s effectiveness in motivating participants to take actions to improve their health, finances and relationships.

AMP has already reached over 6093 older adults across 26 states since 2013. The program is offered in English and Spanish via senior centers, community colleges, community centers and retirement centers in 160+ sites nationwide. Evaluation of pilot deliveries involving 235 participants (without controls) in Virginia found significant pre to post intervention improvements in social connectedness, physical activity levels, healthy eating habits, use of advanced planning, communication with doctor, use of Medicare preventive benefits, medication adherence, participation in evidence-based programs and adoption of other healthy behaviors. In addition, 93% of program participants graduated (completed 7 of 10 classes) [16]. Finally, in other prior uncontrolled evaluation studies, site directors reported they were highly satisfied with the training and technical assistance provided by the National Council on Aging and with the facilitator’s guide, implementation and fidelity guide, participant manual, recruitment material templates and additional tools and templates for program implementation [16,17]. Given these preliminary findings, a more formal evaluation appeared warranted to more definitively establish the evidence for the intervention.

## 2. Materials and Methods

The objectives of the study were to test the effectiveness of AMP in improving overall self-appraisals of health and quality of life, readiness to take positive steps in improving quality of life (activation) and to undertake changes in immediate activities (improved levels of physical activity) and in preparing for the future (advanced care planning), The hypotheses were that there would be significant improvements in all of the described areas.

Given the potential for contamination across conditions if persons were randomly assigned within an implementation site, assignment to conditions was by center where intervention was to be delivered rather than by individual. The study therefore employed a 2 (intervention conditions) × 10 (4 New York City senior/aging network agency centers and 6 upstate senior/aging network centers) × 3 (assessments), nested, partial crossover control group design. In pairs, 2 mostly white population centers in the City, 4 mostly white aging network agencies such as senior centers in Upstate New York, 2 mostly African-American/Black population centers in the City and 2 mostly African-American/Black centers in Upstate New York, all able to support a class of 25–30 participants (284 total) were recruited. The random assignment of pairs of centers to conditions then resulted in a nested design. Experimental group participants received the intervention and control participants received routine center programming comprising self-selected social and educational activities. The control group participants then received the intervention after time two measurement and their outcomes were again measured at Time 3. This resulted in a partial crossover design increasing confidence in findings [18]. 

### 2.1. Intervention

The Aging Mastery Program^®^ (AMP), developed by the National Council on Aging, is designed to be a comprehensive, fun and engaging education and behavior change program for aging well. Central to the AMP philosophy is the belief that modest lifestyle changes can produce big results and that people can be empowered and supported to cultivate health and longevity. Equally important, the program encourages mastery, i.e., developing sustainable behaviors across many dimensions that will lead to improved health, stronger financial security and overall well-being.

The core Aging Mastery curriculum consists of ten classes offered weekly. It is a program that combines education with goal-setting, daily practices and peer support to help participants make meaningful, measureable and enduring changes in the areas of health, finances, life enrichment and advanced planning. The 90-min core sessions, facilitated by trained leaders and delivered by subject matter experts, address Navigating Longer Lives, Exercise and You, Healthy Eating and Hydration, Sleep, Medication Management, Financial Fitness, Advance Planning, Healthy Relationships, Falls Prevention and Community Engagement. Participants are encouraged between classes to make plans related to each topic and to track how they’ve adopted a new healthy behavior or practice. Curriculum materials are provided and all facilitators have received a 3 h training from the developer. More information on the program and curriculum may be found at https://www.ncoa.org/healthy-aging/aging-mastery-program/.

### 2.2. Sample

All participants were 60 and older, both men and women were eligible to participate and confirmation of basic literacy was required. As may be seen in Table 1, the 284 participants were mostly female, on average aged 72 years, primarily White and Black/African-American and had two or more chronic conditions. Also 32% were caregivers. There were no significant differences in the characteristics of the initial experimental and control groups.

### 2.3. Measures

Data was gathered on:

Demographics: Information was gathered on age, sex, race/ethnicity, chronic conditions and caregiving status. Participants were also noted as living in either New York City or in UpState New York. Health and quality of life: Participants were asked to rate their general health and their quality of life, both on five point scales. Patient activation: The 13 item Patient Activation Measure (PAM) was utilized, a scale which has been demonstrated to be a valid (based on concurrence with judge ratings) and highly reliable (alpha: 0.91) measure of a patient’s knowledge, skills and confidence in managing their own health and care and reflecting a developmental model of activation which may be grouped into four categories [19]. Physical activity: The days of week and minutes walked questions taken from the International Physical Activity Questionnaire (IPAQ-L) [20] were completed by all participants. Advance care planning: the nine-item contemplation (scored on a five-point scale) and the 18 item (scored yes/no) subscales for the Advance Care Planning Engagement Survey that measures advanced care planning behavior [21].

Although there was potential given the program content to gather data on other outcome measures these three—activation, walking and advance care planning were selected as they were considered after an initial review of the demographics of senior center participants most likely to capture change over time for the greatest number of participants. 

### 2.4. Protocol Administration:

Both groups completed pre-tests prior to the intervention and the first posttest at ten weeks. Time 1 to Time 2 change in both initial conditions was compared on the measures of interest to see if experimental subjects had significantly larger change in outcomes. After first post-test, the participants in the wait-list control centers received the intervention and they and the original intervention/experimental group participants were followed for an additional ten weeks and completed a third set of measures. The first Post-test measure for control group centers became their baseline measure for the intervention.

### 2.5. Data Analysis

Baseline variables were compared using Student’s *t* and chi square tests. The data for all outcome variables were tested and found to approximate a normal distribution.

To test for the effects of condition, time and Condition × Time interaction effects, we analyzed outcome variables using random effects regression models. Of primary interest in this study was the Condition × Time interactions for the baseline to Time 2 observations. This interaction indicates whether participants receiving the AMP intervention experienced significantly different changes in the outcome variables as compared to those in the wait-list control condition. Because we employed a partial crossover design, it is also of interest to examine time effects and Condition × Time interaction effects for the contrast comparing the baseline to Time 2 period for experimental participants with the Time 2 to Time 3 period for wait-list control group participants. If the support group intervention was effective for both groups of participants, we would expect a significant time effect on this latter contrast but no other significant interaction effects.

Random effects regression models offer several advantages over more traditional repeated measures designs or nonparametric tests [22,23]. The random effects regression model used here included adjustments for first-order autoregressive error terms, which account for whether data collected during follow-up assessment periods were correlated with data collected during the previous time period. Random effects regression models allow all subjects to be included in the analysis. Random subject effects are also included to control for subject-to-subject differences

Analyses were conducted using both SPSS 23 (IBM Corp., Armonk, NY, USA) and SAS (SAS Institute Inc., Cary, NC, USA) and the planned level of significance sought was *p* < 0.05.

## 3. Results

No significant differences in demographics were found between the initial experimental and control groups. Participants in New York City sites were, however, more likely to be Black/African-American and Hispanic/Latino and Upstate New York site participants were a little older. Among participants, approximately 75.6% of participants attended sufficient classes to be considered completers (seven out of ten sessions). Of 291 study participants, 284 (97.6%) completed the baseline protocols. Approximately 30 participants did not complete the second administration and 47 did not complete the third administration. Those who dropped out were more likely to be male at Time 2 and female at Time 3 and also to be older at time three but there were no significant differences between groups as a result of dropping out and those who did drop out were not found to be significantly different on baseline measures as compared to the overall sample. Regardless, an intent-to-treat approach and use of partial data when available, as permitted by Random Effects Regression Models (RERM), helped to correct for any effects of this rate of drop out.

With regard to the primary outcome measures, as can be seen in Table 2, significant interaction effects were found for days walking, minutes walking, information planning and care planning action from baseline to Time 1. An inspection of the means presented in Table 2 reveals that from.

Baseline to Time 1 there was a significantly greater increase in desired activity in all four areas meaning more physical activity on more days and more gathering of information and taking actions related to advanced planning.

Table 3 presents data from the partial crossover design comparing treated participants in both conditions.

As can be seen, there are significant time effects for days walking, minutes walking, information planning and care planning action indicating that intervention had a statistically significant impact in these areas for participants in both conditions supporting the positive outcomes previously reported in Table 2.

## 4. Discussion

A majority of older adults wish to remain in their own homes and communities throughout their aging years [24]. Diminished and declining health and functioning, unavailability of family members [due to death or moving away] and increasing costs make aging in place more difficult [5]. Planning to remain often requires consciously building and seeking to maintain community connections, engaging in health promoting behaviors and actively managing changes in health status [5] AMP is intended to directly address these challenges, offer useful information, begin planning processes and offer experiences likely to increase a sense of self-efficacy [17].

The range of topics covered in the ten weeks on the one hand challenges the ability to find outcomes; few individuals will choose to make changes in every area; and yet the possibility of some change is increased as each person may find an area that is of interest. This evaluation was targeted at understanding if the intervention would result in immediate change (increased physical activity) and future change preparation (advanced care planning) and there were positive findings. There were statistically significant improvements in physical activity (immediate) and in advance care planning activity and readiness (future) as a result of participation in AMP, both important aspects of healthy aging. There was no improvement in measures of overall appraisal of physical health and quality of life or in activation. Given that over 70% had two or more chronic conditions and the types of chronic conditions noted, perhaps improvements in overall appraisals did not occur overall as the challenges faced were not amenable to change over such a short time period. More research is needed to identify circumstances where such change may be possible and where there is targeting of more specific domains of quality of life: material well-being, physical well-being, social engagement and emotional well-being [5]. The low level of activation at baseline may also have meant that capturing change in activation was possible in the timeframe. Here too more research with people with a greater range in baseline activation scores may identify those who may better benefit from AMP in terms of activation. 

There are limitations to the study. Randomization was by pairs of centers rather than by person. However, this had the positive effect of limiting potential contamination of conditions because separation by site meant there was no contact between subjects in different conditions. Also, any limitation is further addressed by the intent to treat methodology as it necessarily means a conservative approach to finding effects, which may also mean that actual effects for those who participated may have been greater. This appears confirmed by the earlier uncontrolled effects found on additional outcomes in the Virginia pilot. However, some level of drop-out is likely in day to day delivery of any program. It is of more interest to the agencies that may adopt AMP that effects appear strong enough, that regardless of drop-outs, the overall effect of this intervention is positive. The purposeful selection of centers in New York City often in challenged communities and in rural and upstate New York on the one hand means that the sample was not representative of all older Americans. However, on the other hand, this purposeful selection did mean that even for challenged participants with multiple chronic conditions, the study achieved positive outcomes.

As scalability is considered, the demonstration of effects for challenged individuals speak to scalability value for participants. That the intervention was successfully implemented in a range of agencies and locations also speaks to scalability value for implementers. What remains to be established is value for payers.

Larger number of participants in future studies will offer more data on subsamples of older adults by age and other characteristics, measures for other outcomes including mental health measures (given some anecdotal reports of improvements) and opportunities to further examine the impact of AMP on outcomes of interest to payers. That said the findings here support that AMP has the potential to be a scalable intervention and future work would then test the range and limits of such scaling. AMP is already demonstrated to be an important addition to the range of interventions being offered to older adults so that they may build their self-management skills, consider critical issues to their current and future well-being and successfully maintain themselves where they would like to live.

As in the earlier uncontrolled studies there were high levels of participation and positive comments from providers. Willingness of funders to pay still needs to be established. Nevertheless, the data on participant attendance and outcomes and positive anecdotal reports from providers do appear to support that AMP demonstrates two of three characteristics known to enhance the possibility of scalability.

## 5. Conclusions

Findings on the Aging Mastery Program support the program’s effectiveness and its value as an evidence-based intervention for older adult programming. Future work will further establish populations where it will be most effective and the potential for additional beneficial outcomes. 

## Figures and Tables

**Table 1:** Demographics (*n* = 284).

Overall		Intervention	Control	
		Mean (SD) %	Mean (SD) %	Mean (SD) %
Age		72.8 (12.29)	73.1 (10.73)	71.9 (11.36)
Sex	Male	11.5%	12.0%	11.0%
	Female	88.5%	-	-
Race/Ethnicity *	Asian	1%	1%	1%
	Black/African American	28.7%	29.3%	27.4%
	Hispanic/Latino	9.1%	9.0%	8.9%
	White	67.5%	67%	68%
	Other	1%	1%	1%
Chronic Conditions	Two or More	72.8%	74%	71%
	Arthritis	38%	38%	37.5%
	Depression/Anxiety	22.3%	21.1%	23%
	Diabetes	22.7%	23%	22.4%
	Heart Disease	14.5%	15%	14.3%
Caregiver		32.1%	31.4%	33.3%

* Adds up to more than 100% as some people checked both Black/African American and Hispanic/Latino.

**Table 2:** Change in participants’ baseline to Time 1.

Baseline	Time 1		F Statistic			*p*-Value
Measure	Mean (SD)	Mean (SD)	Group	Time	Interaction	
General Health						
Treatment	2.65 (1.01)	2.73 (0.93)	23.33	1.43	1.45	
Control	2.64 (1.03)	2.66 (1.02)				
General Quality of Life						
Treatment	8.10 (1.62)	8.19(1.68)	36.89	1.24	1.31	
Control	8.12 (1.61)	8.14 (1.74)				
Patient Activation						
Treatment	39.13 (5.82)	9.55 (5.39)	38.24	2.37	0.67	
Control	39.27 (5.45)	39.20 (5.84)				
Days Walking						
Treatment	3.8 (2.19)	4.44 (1.09)	26.73	8.13	12.78 *	0.04
Control	3.7 (1.89)	3.73 (1.95)				
Minutes Walking						
Treatment	80.75 (44.7)	93.21 (56.9)	85.47	0.04	14.16 *	0.03
Control	82.65 (64.9)	78.34 (83.3)				
Information Planning						
Treatment	4.02 (0.98)	4.34 (0.77)	45.86	3.28	7.34 *	0.04
Control	4.01 (1.23)	4.11 (1.37)				
Care Planning Action						
Treatment	8.03 (5.38)	9.22 (4.89)	40.10	2.92	7.86 *	0.05
Control	7.75 (5.76)	8.01 (5.22)				

* denotes that *p-*Value relates to “interaction”.

**Table 3:** Change in participants after intervention.

Baseline	After Intervention		F			*p-*Value
Measure	Mean (SD)	Mean (SD)	Group	Time	Interaction	
General Health						
Treatment	2.65 (1.01)	2.73 (0.93)	13.73	0.75	0.35	
Control	2.66 (1.02)	2.64 (.97)				
General Quality of Life						
Treatment	8.10 (1.62)	8.19(1.68)	14.89	0.28	1.43	
Control	8.14 (1.74)	8.20 (1.86)				
Patient Activation						
Treatment	19.13 (5.82)	19.55 (5.39)	25.47	1.39	0.60	
Control	19.20 (5.84)	19.63 (6.74)				
Days Walking						
Treatment	3.8 (2.19)	4.44 (1.09)	23.93	8.18 *	0.09	0.04
Control	3.73 (1.95)	4.82 (1.45)				
Minutes Walking						
Treatment	80.75 (44.7)	93.21 (56.9)	26.59	12.15 *	0.16	0.04
Control	78.34 (83.3)	91.64 (97.5)				
Information Planning						
Treatment	4.02 (0.98)	4.34 (0.77)	19.27	9.62 *	1.07	0.04
Control	4.11 (1.37)	4.43 (1.33)				
Care Planning Action						
Treatment	8.03 (5.38)	9.22 (4.89)	17.45	13.97 *	1.21	0.04
Control	8.01 (5.22)	9.47 (3.44)				

* denotes that *p-*Value relates to “time”.

## References

[1] Jacobsen L.A., Kent M., Lee M., Mather M. (2011). America’s aging population. Popul. Bull..

[2] Barbour K.E., Helmick C.G., Theis K.A., Murphy L.B., Hootman J., Brady T.J. (2013). Prevalence of Doctor-Diagnosed Arthritis and Arthritis-Attributable Activity Limitation—United States, 2010–2012. Morb. Mortal. Wkly. Rep..

[3] Ward B.W., Schiller J.S., Goodman R.A. (2014). Multiple chronic conditions among US adults: A 2012 update. Prev. Chronic Dis..

[4] He W., Larson L.J. (2014). Older Americans with a Disability: 2008–2012.

[5] McCallion P., Whitfield K., Baker T. (2014). Aging in Place. Handbook of Minority Aging.

[6] Brady T.J., Murphy L., O’Colmain B.J., Beauchesne D., Daniels B., Greenberg M., House M., Chervin D. (2013). A Meta-Analysis of Health Status, Health Behaviors, and Health Care Utilization Outcomes of the Chronic Disease Self-Management Program. Prev. Chronic Dis..

[7] Diabetes Prevention Program Research Group (2012). Long-Term Safety, Tolerability, and Weight Loss Associated with Metformin in the Diabetes Prevention Program Outcomes Study. Diabetes Care.

[8] McCallion P., Ferretti L.A. (2010). Social work and aging: The challenges for evidence-based practice. Generations.

[9] Lorig K., Holman H.R. (2003). Self-management education: History, definition, outcomes, and mechanisms. Ann. Behav. Med..

[10] McCallion P., Ferretti L.A. (2015). Building Capacity for Self-Management Interventions: The Challenges. J. Nurs. Care.

[11] McCallion P., Ferretti L., Park J., Birkenmaier J., Curley J., Sherraden M. (2013). Financial issues and an aging population: Responding to an increased potential for financial abuse and exploitation. Financial Education & Capability: Research, Education, Policy and Practice.

[12] Rogers E.M. (1962). Diffusion of Innovations.

[13] Hussein T., Plummer M. (2017). Selling Social Change. Stanford Social Innovation Review, Winter. https://ssir.org/articles/entry/selling_social_change.

[14] Rowe J.W., Kahn R.L. (1998). Successful Aging.

[15] Rubenstein R.L., de Medeiros K. (2015). “Successful aging,” gerontological theory and neoliberalism: A qualitative critique. Gerontologist.

[16] National Council on Aging (2016). Report on Preliminary Findings for the Aging Mastery Program.

[17] National Council on Aging (2016). Aging Mastery Program. Manual.

[18] Fortune A.E., Reid W.J. (1998). Research in Social Work.

[19] Hibbert J.H., Stockard J., Mahoney E.R., Tusler M. (2004). Development of the Patient Activation Measure (PAM): Conceptualizing and Measuring Activation in Patients and Consumers. Health Serv. Res..

[20] Graff-Iversen S., Anderssun S.A., Holme I.M., Jenum A.K., Raastad T. (2007). An adapted version of the long International Physical Activity Questionnaire (IPAQ-L): Construct validity in a low-income, multiethnic population study from Oslo, Norway. Int. J. Behav. Nutr. Phys. Act..

[21] Sudore R.L., Stewart A.L., Knight S.J., McMahan R.D., Feuz M., Miao Y., Barnes D.E. (2013). Development and validation of a questionnaire to detect behavior change in multiple advance care planning behaviors. PLoS ONE.

[22] Gibbons R., Hedeker D., Elkin I., Waternaux C., Kraemer H., Greenhouse J., Shea M., Imber S., Sotsky S., Watkins J. (1993). Some conceptual and statistical issues in analysis of longitudinal psychiatric data. Arch. Gen. Psychiatry.

[23] Hedeker D. (1995). Longitudinal Data Analysis. A Workshop, Manual, and Computer Program.

[24] Pynoos J., Caraviello R., Cicero C. (2009). Lifelong housing: The anchor in aging-friendly communities. Generations.

